# IgG-seq identifies immune-reactive enteric bacteria in Crohn’s disease with spondyloarthritis

**DOI:** 10.1080/19490976.2025.2464221

**Published:** 2025-02-13

**Authors:** Grace A. Maldarelli, Maeva Metz, Seun Oguntunmibi, Nancy Tran, Grace Xiang, Dana Lukin, Ellen J. Scherl, Randy S. Longman

**Affiliations:** a Department of Medicine, Division of Infectious Diseases, Weill Cornell Medicine, New York, NY, USA; b Department of Immunology and Microbial Pathogenesis, Jill Roberts Institute for Research in Inflammatory Bowel Disease, Weill Cornell Medicine, New York, NY, USA; c Jill Roberts Center for Inflammatory Bowel Disease, Division of Gastroenterology and Hepatology, Weill Cornell Medicine, New York, NY, USA

**Keywords:** Crohn’s disease, spondyloarthritis, *Mediterraneibacter gnavus*

## Abstract

Joint inflammation is the most common extraintestinal manifestation of Crohn’s disease (CD). Although alterations in the enteric microbiota are described in CD with spondyloarthritis (CD-SpA), it is not known whether distinct taxa serve as markers for clinical subtypes of axial (AxSpA) or peripheral SpA (pSpA) in CD. Moreover, it is not yet known whether these taxa generate a specific systemic IgG response. Here, we sequenced the fecal microbiome from 106 individuals (44 CD, 39 CD-SpA, 14 CD-AxSpA, and 9 healthy controls [HC]). This unique cohort revealed distinct taxonomic compositions of CD and CD-SpA compared to HC and demonstrates that the composition of the CD-AxSpA microbiome is distinct from that of CD-pSpA. Using autologous serum, we identified enteric bacteria recognized by serum IgG and demonstrate differences in the IgG coating index of specific bacterial genera associated with CD-SpA. The IgG coating index of *Mediterraneibacter gnavus* differentiated patients with CD-pSpA and is positively associated with joint disease activity. This work illustrates divergent microbiome compositions in CD-SpA subtypes, as well as the recognition of distinct enteric bacteria by serum IgG with the potential to serve as a marker of joint inflammation in CD.

## Introduction

Joint inflammation is the most common extraintestinal manifestation of Crohn’s disease (CD) and includes both axial (CD-AxSpA) and peripheral (CD-pSpA) spondyloarthritis. Previous studies have observed a strong association between microbial dysbiosis and CD-SpA, supporting the hypothesis that microbiota contribute to systemic inflammation in these cases.^1,2^ Increased IgA coating of *Escherichia coli* in CD-SpA identified a mechanistic role for adherent-invasive *E. coli* in shaping mucosal immunity in SpA.^2,3^ In addition, the studies identified distinct alterations in *Mediterraneibacter gnavus* (formerly *Ruminococcus gnavus*), *Dialister*, and *Parabacteroides* as enriched in the microbiome of individuals with HLA-B27-associated ankylosing spondylitis (AS) compared to those without AS including individuals with inflammatory bowel disease (IBD).^4,5^ HLA-B27 status was further identified as a significant contributor to microbiome composition in these patients.^1^ Although nearly all cases of AxSpA not associated with CD express HLA-B27, it is a less specific and sensitive marker in individuals with CD-AxSpA,^6^ suggesting the potential for alternate contributors to pathogenesis from environmental factors including the microbiome.^7^ Previous studies of AS included limited numbers of HLA-B27-negative individuals with CD, and the specific compositional differences between CD-pSpA and CD-AxSpA microbiomes have been less well studied.

We hypothesize that the underlying link between the enteric microbiota and systemic joint inflammation in CD reflects systemic IgG reactivity to constituents of the intestinal microbiome. While previous reports identified enhanced endogenous IgA recognition of enteric bacteria in CD-pSpA compared to CD,^2^ systemic IgG recognition of microbiota in these patient populations has not been evaluated in CD-pSpA or CD-AxSpA. Innovative work demonstrated the utility of systemic IgG binding in identifying enteric bacteria that can translocate to mesenteric fat and initiate Th17 immunity.^8,9^ The present study aims to analyze the enteric microbiome in individuals with CD with or without peripheral or axial spondyloarthritis. Here, we leverage an approach called IgG-seq to identify enteric bacteria coated by serum IgG and evaluate their potential as diagnostic markers of SpA in CD.

## Results

### Microbiome composition varies by subtypes of SpA in individuals with CD

16S rRNA sequencing was performed on fecal samples from 106 individuals (44 CD, 39 CD-SpA, 14 CD-AxSpA, and 9 healthy controls [HC]) prospectively recruited from the Jill Roberts Center for Inflammatory Bowel Disease at Weill Cornell Medicine. Axial or peripheral SpA was defined by clinical and/or radiographic criteria established by the Assessment of Spondyloarthritis international Society (ASAS).^10^ Joint disease activity was assessed prospectively with the Bath Ankylosing Spondylitis Disease Activity Index (BASDAI). There were no significant differences in age or other demographics between the four groups, and no significant difference in Harvey–Bradshaw Index (HBI) or BASDAI between the CD subgroups (*p* > 0.05, ANOVA). (Table 1).

**Table 1.** Baseline clinical and demographic characteristics.

	HC	CD	CD-SpA	CD-AxSpA
Count	9	44	39	14
Median age (IQR)	35 (28–38)	35 (26–51)	34 (28–51)	37 (27–48)
Sex				
M	4 (44%)	14 (32%)	14 (36%)	4 (29%)
F	5 (55%)	30 (68%)	25 (64%)	10 (71%)
Median HBI (IQR)	–	10 (6–15)	9 (6–13)	8.5 (7–12.25)
Median BASDAI (IQR)	–	–	5.15 (4.1–5.95)	5.8 (4.7–6.6)
HLA-B27 status				
Positive		0 (0%)	1 (3%)	3 (21%)
Negative		22 (50%)	14 (36%)	11 (79%)
Unknown		22 (50%)	24 (62%)	0 (0%)
Current biologic therapy				
Anti-TNFα	–	3	4	0
Anti-IL12/23	–	6	6	0
Anti-Integrin	–	5	2	1

**Figure 1.**
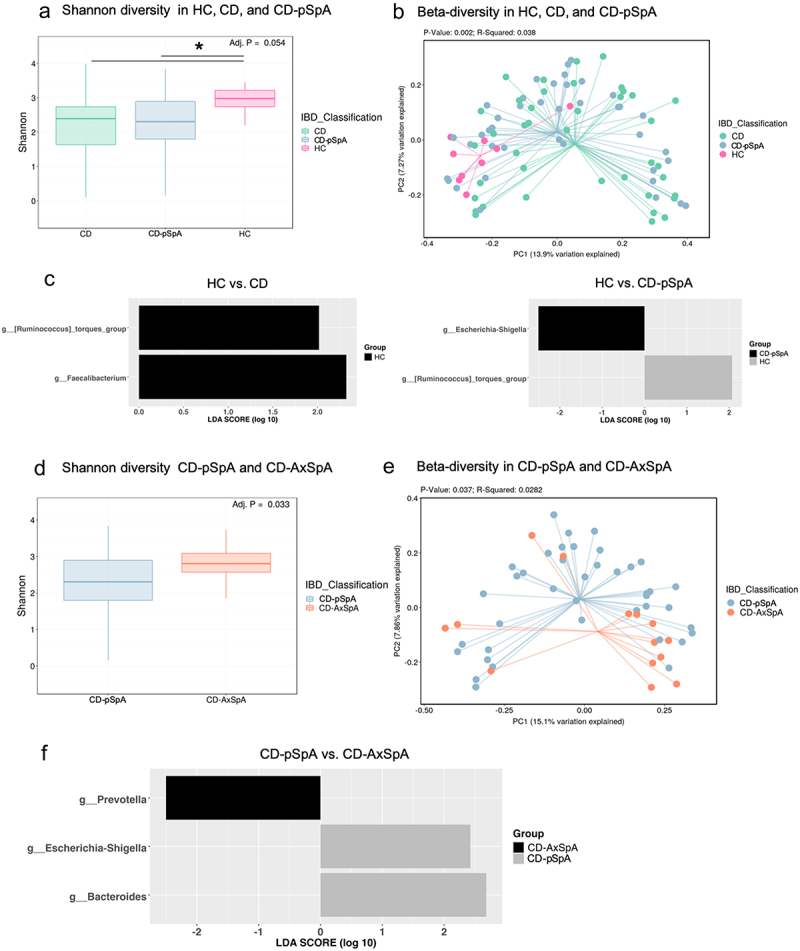
CD subgroups with spondyloarthritis demonstrate divergent microbial diversity and composition. (a) Shannon diversity of samples from CD (*n* = 39), CD-SpA (*n* = 44), and HC (*n* = 9); (*p* = 0.054, Kruskal–Wallis, *p < 0.05). (b) Unweighted Bray–Curtis principal coordinates analysis of microbiome composition of CD, CD-SpA, and HC samples (PERMANOVA *p* = 0.002). (c) LEfSe analysis comparing overrepresented pathways in HC compared to CD, and CD-SpA compared to HC. LEfSe demonstrates no significant enrichment in CD compared to CD-SpA. (d) Shannon diversity of samples from CD-pSpA (*n* = 44) and CD-AxSpA (*n* = 14) (*p* = 0.033). (e) Unweighted Bray–Curtis principal coordinates analysis of microbiome composition of CD-pSpA and CD-AxSpA samples (PERMANOVA *p* = 0.037). (f) LEfSe analysis of CD-pSpA compared to CD-AxSpA.

Analysis of alpha-diversity demonstrated a decreased diversity of the gut microbiome in CD and CD-SpA compared to HC (*p* = 0.054, Kruskal–Wallis; HC–CD, *p* = 0.018, HC–CD-SpA *p* = 0.025), as has been observed previously^11–13^ (Figure 1a). The difference in alpha diversity was coupled with a significant difference in microbiome composition between HC and CD with or without peripheral SpA, as calculated though Bray–Curtis principal components analysis (*p* = 0.002, Kruskal–Wallis) (Figure 1b).

The relative abundance of bacterial genera in each group was evaluated to determine possible drivers of this observed difference (Supplemental Figure 1a). Linear discriminant effect size (LEfSe) analysis demonstrated an enrichment in *Escherichia–Shigella* in CD-pSpA compared to HC, consistent with previous findings^2^ (Figure 1c). Within the CD and CD-pSpA cohorts, 32 individuals were subsequently treated with biologic therapy, but the baseline microbiome did not significantly differ between clinical responders and non-responders (Supplemental Figure 1b).

CD-AxSpA samples were evaluated to determine whether individuals in this specific subgroup develop a distinct microbial signature compared to CD-pSpA. Of note, 11 of 14 individuals in this group were HLA-B27 negative. In comparing the CD-AxSpA samples to the CD-SpA samples, a significant difference was found in both Shannon diversity (*p* = 0.033) (Figure 1d) and in beta-diversity (*p* = 0.037) between these two groups (Figure 1e). This difference is present despite similar joint and intestinal disease activity scores measured by median BASDAI and HBI, respectively, in the two groups (Table 1). LEfSe analysis demonstrates enrichment of *Prevotella* in CD-AxSpA relative to CD-pSpA, and *Escherichia–Shigella* and *Bacteroides* enrichment in CD-pSpA compared to CD-AxSpA (Supplemental Figure S1C and Figure S1F).

### IgG-seq reveals systemic recognition of enteric microbiota in CD-SpA

**Figure 2.**
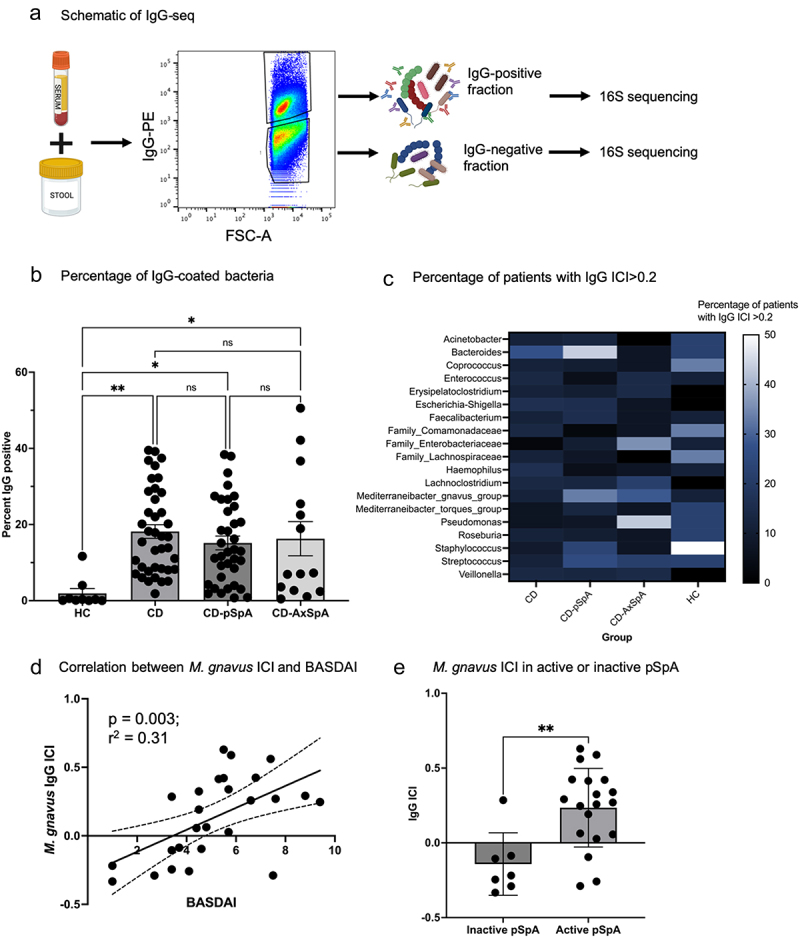
IgG-seq identifies enteric bacteria recognized by serum IgG. (a) Schematic of IgG-seq. Patient stool and serum samples are incubated, stained for IgG binding, and sorted into IgG-positive and IgG-negative fractionsfor 16S sequencing. (b) Overall percentage of IgG coating of bacteria by flow cytometry in CD, CD-pSpA, CD-AxSpA, and HC (*p < 0.05; **p < 0.01; ns, not significant; one-way ANOVA). (c) Genus *Pseudomonas* is recognized by 42% of CD-AxSpA patients compared to 6.8% of CD and 7.7% of CD-pSpA patients. *Mediterraneibacter gnavus*is highly recognized in 33% of CD-pSpA patients but only 11% of CD patients. Given different group sizes, percentage of patients per group (rather than number of patients per group) is shown. (d) Linear regression of *M.* *gnavus* IgG ICI compared with joint disease activity as measured by BASDAI. (e) *M. gnavus* IgG ICI in CD-pSpA patients with active SpA compared to inactive SpA (**p < 0.01). Error bars represent standard error of the mean.

Given the unique extraintestinal symptom profile of this cohort and the initial results demonstrating compositional differences in the microbiome in CD-SpA subtypes, we pursued the hypothesis that enteric bacteria drive systemic immunity in the setting of CD-pSpA and CD-AxSpA. We reasoned that evidence of this interaction can be exploited by determining autologous serum IgG coating of fecal bacteria to identify possible contributors to the development of extraintestinal manifestations of CD. Previous studies have used endogenous IgA coating of bacteria to identify contributors to intestinal mucosal immunity, in a strategy called IgA-seq.^2,14,15^ More recent studies have used circulating IgG recognition of enteric bacteria to identify taxa that translocate through the intestinal mucosa.^16^ To identify enteric bacteria with systemic IgG reactivity in subjects with extraintestinal manifestations of CD, we adapted this approach using autologous serum and stool (rather than using a standardized set of bacteria), a protocol referred to here as IgG-Seq (Figure 2a).

IgG-seq was conducted on paired stool and serum samples from the full cohort of CD, CD-pSpA, CD-AxSpA, and HC individuals. To evaluate the possible correlation between IgG coating and clinical phenotype, we analyzed the overall percentage of bacteria that were recognized by IgG, and found that both CD and CD-SpA demonstrate an increase in IgG coating compared to HC (Figure 2b). To evaluate endogenous IgG binding, we also sorted samples not incubated with serum. While low levels of endogenous IgG coating were present in all CD groups as expected due to intestinal inflammation,^17^ negligible endogenous IgG coating is observed in HC (Supplemental Figure 2a, b).

To assess differences in IgG coating of individual taxa across patients, we applied a normalized index of IgG enrichment across the patient cohort, called immunoglobulin coating index (ICI), which calculates the genus frequency in the IgG-positive compared to the IgG-negative fractions using a log-normalized equation.^14^ To determine which of these bacterial genera are the most dominant and relevant taxa to discriminate between clinical phenotypes and to ensure generalizability, we identified genera with an ICI > 0.2 in at least 10% of samples. Using this approach, we observed increased IgG ICI of *Bacteroides* in 44% of individuals with CD-pSpA compared to 27% with CD, 7% with CD-AxSpA, or 22% of HC (Figure 2c). In contrast, 55% of HC have elevated IgG ICI of *Staphylococcus*. Additionally, an increase in IgG ICI for *Pseudomonas* is observed in individuals with CD-AxSpA (42% of individuals with CD-AxSpA compared to 22% of HC); however, the IgG ICI of *Pseudomonas* does not correlate with joint disease activity as measured by BASDAI (Supplemental Figure 2c). IgG coating of *M. gnavus* is observed specifically among individuals with CD-pSpA and CD-AxSpA compared to other groups (33% in CD-pSpA and 29% in CD-AxSpA, compared to 11% in CD and HC). Recognition of *M. gnavus* as measured by IgG ICI is associated with BASDAI among individuals with CD-pSpA (*p* = 0.003, R-squared = 0.31) (Figure 2d). Furthermore, individuals with active SpA (BASDAI >4) have a significantly higher *M. gnavus* ICI compared to those with inactive SpA (Figure 2e). Generation of receiver operating characteristic (ROC) curves using the IgG ICI for *Pseudomonas*, *Enterobacteriaceae*, and *M. gnavus* discriminates CD-pSpA from CD-AxSpA with an area under the curve (AUC) of 83% (95% CI: 70–96%) (Supplemental Figure 2d). These marker taxa also discriminate clinical responder from non-responders to CD induction therapy (Supplemental Figure 2e).

## Discussion

Using a unique cohort of CD with and without axial or peripheral SpA, we identify differences in the composition of the intestinal microbiome between CD-pSpA and a majority-HLA-B27-negative CD-AxSpA population. Similar to our results, previous work identified an expansion in *M. gnavus* in an HLA-B27-positive AxSpA cohort, which correlated with disease activity,^1^ suggesting a potential broader role for this taxa independent of HLA-B27. Strains of *M*. gnavus have been causally associated with the development of lupus nephritis, through the production of lipoglycan.^18,19^ Even in the absence of HLA-B27, the microbiome of CD-AxSpA diverges from that of CD-pSpA, demonstrating enrichment of *Prevotella* in individuals with CD-AxSpA.^20^ Enrichment of *Prevotella* in the fecal microbiome was previously seen in individuals with pre-clinical and new onset stages of rheumatoid arthritis, independent of shared HLA epitopes.^20,21^ These data are consistent with a contribution of *Prevotella* enrichment to inflammation even in the absence of HLA-restricted disease, but additional validation cohorts comparing HLA-B27 negative and positive CD-AxSpA are needed. Finally, this study expands previous work from our lab demonstrating an increase in *Enterobacteriaceae* abundance in CD-pSpA compared to CD.

Beyond compositional differences in the fecal microbiome, we identify differences in serum IgG coating of enteric bacteria in the SpA subgroups with CD compared to HC. Of note in our HC population, the genus *Staphylococcus* was highly coated. Although this genus includes noted pathogens *S. aureus* and *S. lugdunensis*, other staphylococcal species are common skin commensals, and IgG recognition may reflect homeostatic immunity. A study of serum samples from the Mayo clinic similarly demonstrates that antibodies to *S. aureus* antigens are more prevalent in HC compared to individuals with CD or UC.^22^ We also observed an increased IgG coating index of *Bacteroides* in CD compared to HC. This finding of increased *Bacteroides* recognition in CD may reflect variable associations of different *Bacteroides* species with IBD.^17,23^

Biomarkers of joint disease activity in CD-SpA are limited. The IgG-seq results reported here demonstrate that *M. gnavus* has a higher IgG coating index in a population with CD-SpA compared to CD alone, and the degree of recognition is positively correlated with joint inflammation. *M. gnavus* has been identified as an organism capable of translocation from the colon into the mesenteric fat. Consistent with the possibility that intestinal inflammation enables enhanced translocation, serum from subjects with IBD demonstrated increased recognition of *M. gnavus* in a surrogate fecal community compared to sera from HC.^16^ *M. gnavus* encodes a number of proinflammatory molecules, including lipoglycan and glucorhamnan, as well as a superantigen-like protein, IbpA, which may contribute to its antigenic properties.^19,24,25^ In addition, M. *gnavus* also expresses strain-specific antigens capable of interacting with mucosal surface receptors.^26^ Consistent with our results, previous work showed that IgG binding enrichment of *M. gnavus* did not distinguish HC from IBD.^27^ Increased recognition of *M. gnavus* specifically in individuals with CD-SpA from this cohort provides further evidence for closer study of this organism in the setting of CD-SpA and other autoimmune disease. Additional validation cohorts are needed to test the clinical utility of *M. gnavus* seroreactivity as a biomarker of SpA activity in CD.

There are several limitations of the current study. Given previous work suggesting the higher efficacy of anti-TNFα compared to anti-IL12/23 therapy for SpA symptoms,^28^ further work is needed to define the potential medication-specific impact and to assess longitudinal tracking of specific strains associated with joint disease activity. While this work demonstrates that bacterial taxa enriched in IgG coating can discriminate CD-SpA subtypes and response to CD therapy, our study is limited to a single cohort. Additional validation cohorts with both HLA-B27-negative and -positive CD-AxSpA are needed to assess the diagnostic value of these findings. Finally, functional follow-up studies will help to elucidate the potential immunologic mechanisms by which *M. gnavus* contributes to CD-SpA.

## Methods

### Patient cohort

A prospective cohort of CD patients and HC was evaluated for spondyloarthritis (SpA) at the Jill Roberts Center for IBD (JRC IBD) under IRB-approved protocol (1806019340 and 1103011578) at Weill-Cornell Medical College. Subjects between the ages of 18–80 with biopsy-proven ileal or ileo-colonic (CD with or without clinical evidence of peripheral or axial SpA (according to the ASAS guidelines)^29^ signed informed consent to participate in the research. Healthy control subjects without IBD or active intestinal symptoms were recruited from our GI practice. All subjects were free of other rheumatic disease, off antibiotics (including sulfasalazine) for at least 8 weeks at enrollment, and had an intact colon and ileocecal valve. All patients completed the BASDAI – a clinically validated exam for disease activity in AS.^29^ The clinical response to therapy was defined by a reduction in the HBI score by 3 or greater. Fresh fecal samples and serum were obtained and stored at −80°C until further analyses.

### IgG-seq

For IgG-seq, stool was filtered and incubated with autologous serum prior to staining with PE-rat anti-human IgG (BioLegend, San Diego, CA). Given intra-patient differences, IgG-PE gating was modified slightly for each individual sample to account for these differences. For endogenous IgG coating experiments, samples were not incubated with autologous serum prior to sorting. Samples were sorted into IgG-positive and -negative fractions using a BD FacsMelody sorter (Becton-Dickinson, Franklin Lakes, NJ). A minimum of 10,000 events were sorted for each fraction. DNA was extracted using a Qiagen PowerSoil kit (QIAGEN, Germany). Aliquots from samples drawn prior to sorting were also extracted. 16S sequencing of these samples was conducted through the Weill Cornell Medicine Microbiome Core Facility; samples were prepared using the Earth Microbiome Project protocol^30^ and sequenced on Illumina MiSeq (Illumina, San Diego, CA). Sequences were processed with FastQC, and QIIME2 via a custom pipeline.^31^ Amplicon sequence variants (ASVs) were mapped to an optimized version of the SILVA database. One CD sample did not pass quality control for sequencing and was not used for further analysis.

### Sequence processing and statistical analysis

Merged biom and mapping files were imported into R using phyloseq.^32^ Shannon diversity and unweighted Bray–Curtis plots were generated using ATIMA.^33^ LEfSe was conducted using the lefser package for R^34^ (https://github.com/waldronlab/lefser), and LEfSe results were graphed using ggplot2. A normalized IgG ICI was calculated using the IgAScores package for R.^14^ RROC plots were generated using the pROC package for R.^35^

Highly recognized genera were selected using ICI > 0.2 as a cutoff; an ICI of 0.2 represents a genus with 0.1% relative abundance in the IgG-positive fraction and a relative abundance of 0.01% in the negative fraction, a one-log increase in relative abundance in comparing the fractions. Differences between groups were compared using Mann–Whitney (for two categories) or Kruskal–Wallis (for more than two categories) non-parametric tests. The Benjamini–Hochberg correction was used to correct for false discovery rate.

## Supplementary Material

Supplemental Material

## Data Availability

The raw sequencing data used for this study are publicly available via the NCBI Sequence Read Archive repository under accession number SUB14844119.
