# Supplementary figures and images for: IgG-seq identifies immune-reactive enteric bacteria in Crohn’s disease with spondyloarthritis
Source: Gut Microbes. 2025 Feb 13;17(1):2464221. doi: 10.1080/19490976.2025.2464221 (PMC11834481; doi:10.1080/19490976.2025.2464221)

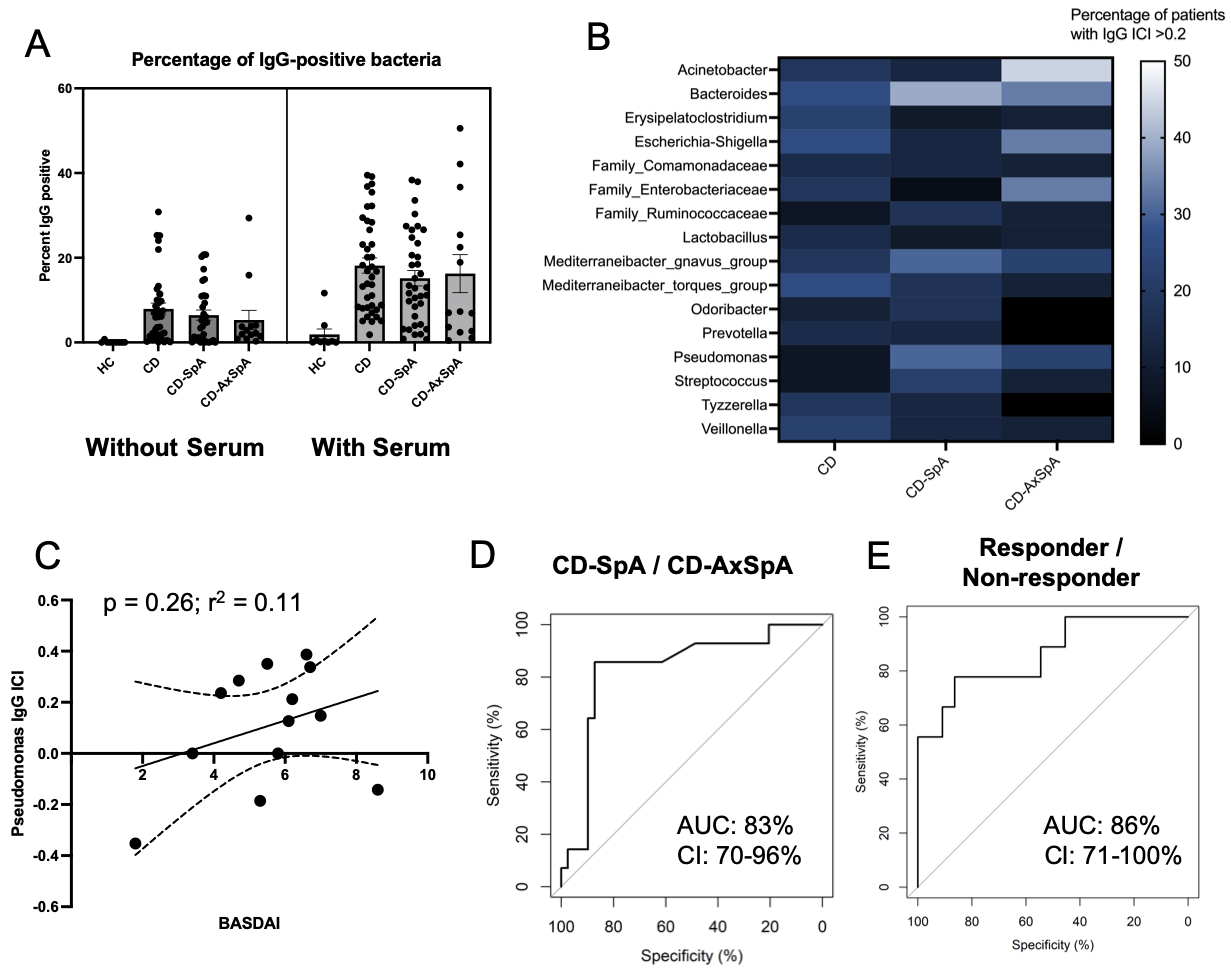

Supplement: Supplemental Material [file KGMI_A_2464221_SM7289.zip › supplemental_figure_2_01312025.png]

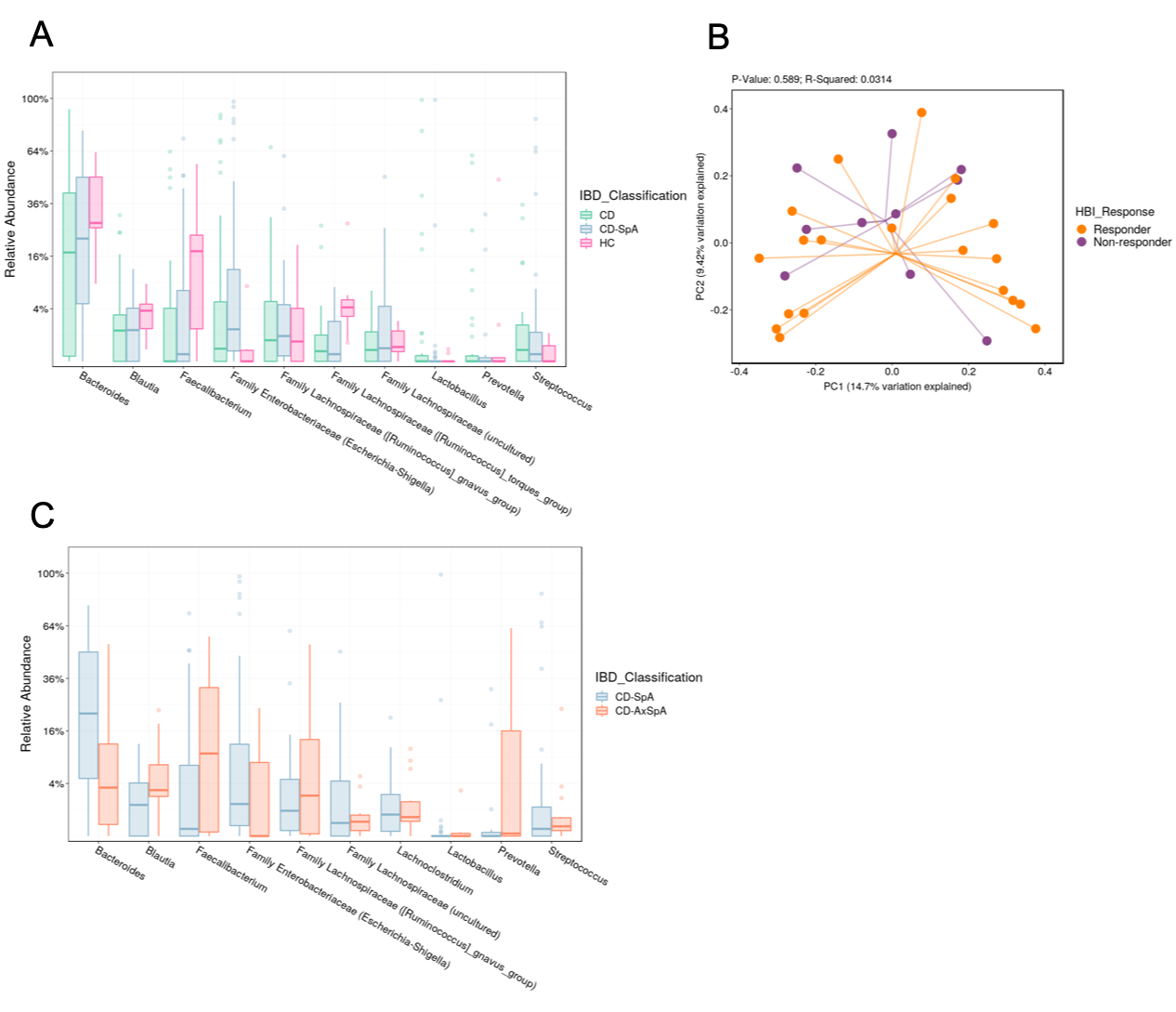

Supplement: Supplemental Material [file KGMI_A_2464221_SM7289.zip › supplemental_figure_1_01312025.png]
